# Detection of Creutzfeldt-Jakob disease prions in skin: implications for healthcare

**DOI:** 10.1186/s13073-018-0536-3

**Published:** 2018-03-26

**Authors:** Akin Nihat, Simon Mead

**Affiliations:** 1 National Prion Clinic, The National Hospital for Neurology and Neurosurgery, University College London Hospitals NHS Foundation Trust, Queen Square, London, WC1N 3BG UK; 2 Medical Research Council Prion Unit at University College London (UCL), Institute of Prion Diseases, London, W1W 7FF UK

## Abstract

Evidence has recently been reported of prion seeding activity in skin tissue from patients with sporadic Creutzfeldt-Jakob disease (sCJD). This is relevant information for infection control measures during surgery. The work uses very sensitive prion assays now available for medical research, and may soon be adapted to related neurodegenerative disorders.

## Prion diseases

Prion diseases are a group of incurable neurodegenerative disorders marked by the accumulation of misfolded forms of the normal cellular prion protein (PrP). The concept of a proteinaceous pathogen or ‘prion’ responsible for the transmissibility of the diseases, although initially controversial, has become an influential concept in neurodegeneration research, but it is still unclear exactly which structures of abnormal PrP behave as prions. The most common human prion disease, sCJD, occurs at random in the population. However, this disease group is notorious for acquired forms: zoonotic variant CJD (vCJD) arises following dietary exposure to bovine spongiform encephalopathy (BSE) prions; and iatrogenic CJD arises from exposure to prions as a result of a medical procedure. Procedures known to have caused iatrogenic CJD include the use of cadaveric growth hormone, dura mater grafts, and, especially pertinent to a discussion about prions in skin, surgical instruments. Extensive measures are taken to prevent patient exposures to prions during medical or surgical procedures in those asymptomatically infected, or with early but unrecognised symptoms. Acquired prion diseases are very rare. In 2017, 113 deaths were recorded in the UK from definite or probable prion diseases, but none of these cases were thought to be acquired (National CJD Research and Surveillance Unit data, https://www.cjd.ed.ac.uk/).

## Evidence for prions in skin

Animal bioassay is the only method to definitively demonstrate prion infectivity, but these are expensive and time-consuming experiments. Many factors affect the efficiency of transmission in an experiment: inoculation route, PrP expression level of the inoculated animal, prion strain (comparable to virus strain), and the degree of homology in the primary sequence of PrP between host and inoculum. By varying these factors, experiments can be designed to optimise sensitivity, or model medical situations more realistically. Recently, Orrú and colleagues [1] demonstrated, for the first time, gold-standard evidence for the presence of human prions in skin, using a transmission study tuned to be sensitive: by intracerebral inoculation of inocula made from the skin of two patients with CJD, into mice that were engineered to express the human form of PrP.

Surrogate methods were also used to infer the presence of prions in skin tissue. These methods do not directly measure prion titres or infectivity but are fast, inexpensive, and sensitive. The disease process generates a multitude of abnormal forms of prion protein that can be either infectious or non-infectious. Many abnormal forms of prion protein have distinct biochemical properties such as relative protease resistance and staining properties of amyloid protein aggregates, and can be detected by histology or partial protease digestion and Western blotting. Orrú et al. [1] found only a faint PrP-immunoreactive band by Western blot from one of five sCJD patients despite using enhanced detection techniques. This finding is broadly consistent with previous studies [2].

More significantly, Orrú et al. [1] employed in vitro prion ‘seeding’ assays, which are able to detect miniscule amounts of disease-associated prion protein (~femtograms). The real-time quaking-induced conversion (RTQuIC) assay exploits the ability of disease-associated prion protein to template the misfolding of recombinant PrP through repeated cycles of mechanical agitation intended to break apart the forming amyloid. In a blinded analysis, Orrú et al. [1] demonstrated by RTQuIC the presence of prion protein amyloid in at least one of three skin samples from all 23 CJD patients with either vCJD or sCJD, but not in skin from any non-CJD control individuals. The concentrations of RTQuIC seeding activity were 1000- to 100,000-fold lower in skin than brain tissue from the same patient. These RTQuIC results are meaningful and quantitative, but a caveat is that the assay can amplify non-infectious prion protein aggregates; therefore, the assay results are a surrogate for prion titre only. Analysis of a larger dataset that includes samples from healthy elderly individuals and/or those with conditions that might be mistaken for CJD would increase confidence in the specificity of this approach for CJD diagnosis.

Variant CJD infection has been transmitted by blood or blood product transfusion on at least five occasions, which has led to restrictions from the blood donor pool for groups deemed to be at high risk. Approaches to detect variant CJD prions in blood samples using protein misfolding cyclic amplification (PMCA) have been described in two recent papers [3, 4]. PMCA disrupts aggregates using sonication rather than shaking, requires a biological source for normal PrP rather than a recombinant protein, and uses Western blot as a readout. In one study, blood from two donors who later developed vCJD also demonstrated the presence of prions by PMCA [4]. Data from these studies provide a key step toward the validation of PMCA technology as a blood-based diagnostic test for vCJD and support its potential for detecting pre-symptomatic patients. However, the prospect of using prion detection assays to ensure the safety of blood transfusion is mitigated by several technical challenges that have yet to be overcome. Thankfully, there have been no patients diagnosed with blood-transmitted variant CJD for 10 years.

## Implications for infection control and diagnosis

Reports of prion transmissions, whether experimental or observational, can sometimes result in excessive media attention and misunderstandings. On occasion, notably during the BSE crisis, media attention was justified and major policy decisions needed to be taken. Our role as research scientists is to carefully discuss the findings and limitations of our results, even if not fully conclusive, with the public and policymakers. Orrú and colleagues [1] discuss their results responsibly; they emphasise that prion disease is not known to be transmitted via casual skin–skin contact, but they highlight the potential for iatrogenic transmission from this tissue. They also accept that the extreme sensitivity of the assays and methodologies used, and low RTQuIC titres in skin relative to brain, makes the interpretation of these findings in real-life infection control scenarios ambiguous. Further animal bioassay studies of skin from patients with sCJD may help to clarify the extent of infectivity.

Over 40 years ago, the demonstration of iatrogenic neurosurgical transmission of CJD and the known resistance of prions to standard decontamination methods prompted epidemiological studies of surgery and risk of CJD [5]. Most studies adopted a case–control methodology to identify patients diagnosed with CJD and retrospectively review their surgical histories compared with matched controls. The results are inconclusive, which is perhaps unsurprising in view of the inherent potential for selection bias, recall bias, and that surgery may be carried out to investigate unrecognised early symptoms of CJD [5, 6].

What about CJD diagnosis? Skin biopsy samples in the study by Orrú et al. [1] were obtained mostly from deceased patients. It will be important to ascertain the chronology; that is, whether prions accumulated as an early or late feature of the disease. An earlier study by Glatzel et al. [2] demonstrated that sCJD patients with the highest level of abnormal PrP deposition in spleen and skeletal muscle also had atypical forms and the longest disease duration. Abnormal PrP has also been detected using RTQuIC of material obtained by brushing the olfactory mucosa in sCJD [7], and by adaptation of a blood-based assay to urine [8]. However, although these findings are insightful, the most reliable method of CJD diagnosis is RTQuIC assay using cerebrospinal fluid obtained by lumbar puncture and magnetic resonance brain imaging; both of these techniques are sensitive and highly specific and are a pre-requisite in the work-up of patients suspected to have CJD to exclude other conditions [9].

## Conclusions and perspectives

Abnormal PrP amplification technologies are incredibly sensitive assays that provide evidence for a wider tissue distribution for prions in sCJD and rapid detection in individual patients. Whether these developments will translate into improved infection control measures is a much more complicated question, as it is very hard to find evidence for ongoing person–person transmission with surgical instruments or blood–blood product transfusion. This may be because transmissions are not currently occurring in healthcare settings, or a consequence of the challenges of epidemiological investigation of a rare disorder with potentially very long incubation periods. In this context, any new infection control measures will need to be practical and proportionate.

An increasing body of experimental and observational evidence suggests that more common neurodegenerative diseases, such as Alzheimer’s and Parkinson’s diseases, share fundamental mechanistic similarities with prion disease [10]. Whilst these similarities were proposed following animal transmission experiments and the apparent spreading of protein pathologies in the brain, recent findings raise the possibility of iatrogenic amyloid-beta cerebral amyloid angiopathy in specific circumstances that parallel the experience of acquired prion diseases [11]. This should neither be surprising nor alarming news. In this respect, recent results illustrate the potential of tools developed for prion research for the wider field of neurodegeneration and encourage their adaptation to other misfolded proteins.

## Abbreviations

BSE: Bovine spongiform encephalopathy
CJD: Creutzfeldt-Jakob disease
PMCA: Protein misfolding cyclic amplification
PrP: Prion protein
RTQuIC: Real-time quaking-induced conversion assay
sCJD: Sporadic Creutzfeldt-Jakob disease
vCJD: Variant Creutzfeldt-Jakob disease

## References

[1] Orrú CD, Yuan J, Appleby BS, Li B, Li Y, Winner D, et al. Prion seeding activity and infectivity in skin samples from patients with sporadic Creutzfeldt-Jakob disease. Sci Transl Med. 2017; 10.1126/scitranslmed.aam7785. 10.1126/scitranslmed.aam7785 PMC5744860 29167394

[2] Glatzel M, Abela E, Maissen M, Aguzzi A (2003). Extraneural pathologic prion protein in sporadic Creutzfeldt-Jakob disease. N Engl J Med.

[3] Concha-Marambio L, Pritzkow S, Moda F, Tagliavini F, Ironside JW, Schulz PE (2016). Detection of prions in blood from patients with variant Creutzfeldt-Jakob disease. Sci Transl Med.

[4] Bougard D, Brandel JP, Belondrade M, Béringue V, Segarra C, Fleury H (2016). Detection of prions in the plasma of presymptomatic and symptomatic patients with variant Creutzfeldt-Jakob disease. Sci Transl Med.

[5] Lopez FJG, Ruiz Tovar M, Almazan-Isla J, Alcalde-Cabero E, Calero M, Pedro-Cuesta J. Risk of transmission of sporadic Creutzfeldt-Jakob disease by surgical procedures: systematic reviews and quality of evidence. Euro Surveill. 2017; 10.2807/1560-7917.ES.2017.22.43.16-00806. 10.2807/1560-7917.ES.2017.22.43.16-00806 PMC5718390 29090678

[6] de Pedro CJ, Ruiz TM, Ward H, Calero M, Smith A, Verduras CA (2012). Sensitivity to biases of case-control studies on medical procedures, particularly surgery and blood transfusion, and risk of Creutzfeldt-Jakob disease. Neuroepidemiology.

[7] Bongianni M, Orru C, Groveman BR, Sacchetto L, Fiorini M, Tonoli G (2017). Diagnosis of human prion disease using real-time quaking-induced conversion testing of olfactory mucosa and cerebrospinal fluid samples. JAMA Neurol.

[8] Luk C, Jones S, Thomas C, Fox NC, Mok TH, Mead S (2016). Diagnosing sporadic Creutzfeldt-Jakob disease by the detection of abnormal prion protein in patient urine. JAMA Neurol.

[9] Rudge P, Hyare H, Green A, Collinge J, Mead S. Imaging and CSF analyses effectively distinguish CJD from its mimics. J Neurol Neurosurg Psychiatry. 2017; 10.1136/jnnp-2017-316853. 10.1136/jnnp-2017-316853 PMC5909756 29142140

[10] Jucker M, Walker LC (2013). Self-propagation of pathogenic protein aggregates in neurodegenerative diseases. Nature.

[11] Jaunmuktane Z, Mead S, Ellis M, Wadsworth JD, Nicoll AJ, Kenny J (2015). Evidence for human transmission of amyloid-beta pathology and cerebral amyloid angiopathy. Nature.

